# Bleeding pattern and consumption of factor VIII concentrate in adult patients with haemophilia A without inhibitors in the Czech Republic between 2013 and 2021 (Czech National Haemophilia Programme registry data)

**DOI:** 10.1007/s00277-023-05453-6

**Published:** 2023-09-23

**Authors:** Gabriela Romanová, Petr Smejkal, Petra Ovesná, Eva Drbohlavová, Petr Dulíček, Zdeňka Hajšmanová, Antonín Hluší, Radka Hrdličková, Jana Ullrychová, Ivan Vonke, Jan Blatný, Zuzana Čermáková, Ester Zápotocká, Miroslav Penka

**Affiliations:** 1 Department of Internal Medicine, Haematology and, Oncology University Hospital Brno, Brno, Czech Republic; 2 Faculty of Medicine, Masaryk University, Brno, Czech Republic; 3 Department of Clinical, Haematology University Hospital Brno, Brno, Czech Republic; 4 Department of Laboratory Methods Faculty of Medicine, Masaryk University, Brno, Czech Republic; 5 Institute of Biostatistics and Analyses, Ltd., Brno, Czech Republic; 6 Department of Clinical Haematology, Regional Hospital Liberec, Liberec, Czech Republic; 7 IVth Department of Internal Medicine-Haematology, University Hospital in Hradec Králové, Medical Faculty in Hradec Kralove, Hradec Králové, Czech Republic; 8 Charles University in Prague, Prague, Czech Republic; 9 Department of Haematology, Institute of Clinical Biochemistry and, Haematology University Hospital Pilsen, Pilsen, Czech Republic; 10 Department of Haemato-Oncology, Faculty of Medicine and Dentistry, Palacky University and University Hospital Olomouc, Olomouc, Czech Republic; 11 Blood Centre University Hospital Ostrava, Ostrava, Czech Republic; 12 Department of Clinical Haematology Krajska Zdravotní a.S., Masaryk Hospital Ústí Nad Labem, Ústí Nad Labem, Czech Republic; 13 Clinical Haematology Hospital České Budějovice, České Budějovice, Czech Republic; 14 Department of Paediatric Haematology and Biochemistry, University Hospital Brno, Brno, Czech Republic; 15 Masaryk University, Brno, Czech Republic; 16 Department of Paediatric Haematology and Oncology, University Hospital Motol, Prague, Czech Republic; 17 2nd Medical Faculty, Charles University, Prague, Czech Republic

**Keywords:** Haemophilia, Bleeding, Prophylaxis, On-demand, Consumption of factor VIII/IX concentrate, CNHP (the Czech National Haemophilia Programme), EHL (extended half-life)

## Abstract

The manuscript provides an overview of treatment and its changes in adult patients with haemophilia A without inhibitors in the Czech Republic between 2013 and 2021 using data from the registry of the Czech National Haemophilia Programme (CNHP). Over a 9-year period, we focused on the reduction in the annual bleeding rate (ABR), joint bleeding rate (AJBR) and factor VIII consumption when patients with severe haemophilia A switched from on-demand treatment to prophylaxis. The ABR and AJBR include both patient-reported home treatment and treated hospitalisation episodes. All adult patients with severe haemophilia A were categorised into three groups according to the therapeutic regimen. The first group was patients on prophylaxis during the follow-up period, the second group consisted of patients on on-demand treatment, and the third group was patients who received both treatment regimens during follow-up. With an increase in the proportion of patients with severe haemophilia A on prophylaxis from 37 to 74% between 2013 and 2021, the ABR for all patients with severe haemophilia A decreased approximately 6.9-fold, and the AJBR decreased 8.7-fold. Expectedly, the factor consumption increased by approximately 68.5%. In the group of patients with severe haemophilia A who had switched from an on-demand to a prophylactic regimen, the total number of bleeding events decreased 3.5-fold, and the number of joint bleeding episodes decreased 3.9-fold. Factor VIII consumption increased by 78.4%. Our study supports a previously reported positive effect of prophylaxis on bleeding control. We believe that the substantial improvement in ABR justifies the increased treatment costs.

## Methods

Nationwide outcome/reporting of the number of bleeding episodes and consumption of factor VIII/IX concentrates has been available in the Czech National Haemophilia Programme (CNHP) registry since 2012. Prior to that date, only paediatric centres were involved. Although the CNHP register has been maintained since 2012, the number of (joint) bleedings has been recorded since 2013. The CNHP registry provides data for the last 10 years from all haemophilia treatment centres (except for one) treating adult and paediatric patients with congenital bleeding disorders (Comprehensive Care Centres (CCC) and Haemophilia Treatment Centres (HTC)) in the Czech Republic [1]. Reports from the CNHP registry [https://www.cnhp.cz/index.php?pg=registr—vysledky] are published annually in the Czech Republic and provide information on the total number of patients treated for haemophilia, frequency and bleeding episodes (total and joint), consumption of specific factor VIII/IX concentrates, and surgical or other invasive procedures and allow us to estimate costs associated with the treatment of haemophilia [2]. All study participants signed an informed consent form according to the World Medical Association (WMA) Declaration of Helsinki [3].

The aim of this analysis was to describe the population of adults with haemophilia A without inhibitors, involving analysis of available data from the CNHP registry for the whole period. We focus on trends in the number of bleeding episodes (joint and total) based on the introduction of mainly tertiary prophylaxis and the gradual introduction of new types of factor VIII/FIX concentrates (EHLs). Another goal of this analysis was to compare the effect of prophylaxis in patients who changed their treatment regimen during a specified period. Since haemophilia A with inhibitors includes a specific group of patients, we excluded patients with a current inhibitor from the aforementioned group.

## Statistical methods

Standard descriptive statistics were used for a summary of patients’ characteristics and treatment outcomes—annual bleeding rate (ABR), joint ABR (AJBR) and FVIII consumption in particular years. All adult patients with severe haemophilia A were categorised into three distinct groups according to their treatment regimen: (i) patients on permanent prophylaxis (PX) within the whole period; (ii) patients with on-demand (OD) treatment; and (iii) patients with both treatment regimens recorded during their follow-up (temporary prophylaxis, i.e. years in which a patient was switched from OD to PX or vice versa were excluded). Characteristics and outcomes were annualised and compared between the on-demand and prophylaxis groups using the Wilcoxon rank sum test. The effect of prophylaxis on ABR and AJBR was further adjusted for age using a negative binomial generalised linear mixed-effects model (GLMM) for count data to manage the correlation amongst patients and random effects of patients. In the group of patients with both regimens recorded, the Wilcoxon signed-rank test with continuity correction was applied to compare their treatment outcomes. All analyses were performed in R software (using the lme4 package for GLMM), and tests were performed as two-sided on a significance level alpha = 0.05.

## Results

Between 2012 and 2021, the registry collected data on a total of 669 adult patients with haemophilia A, 245 of whom had severe disease.

### Group of all adult patients with haemophilia A in the CNHP registry

As part of this analysis, we focused on adult patients with haemophilia A who were followed between 2012 and 2021 (Table 1). Data from 358 adult patients with haemophilia A in the registry at the beginning of the follow-up period increased to 470 by 2021. Over the years, there were an increasing number of patients with mild haemophilia A in the registry. In 2012, the group of patients with mild haemophilia accounted for 45%, and in 2021, their proportion increased to 56%. Moderate haemophiliacs at the beginning of the follow-up accounted for 11%, and by the end of the follow-up period, this percentage decreased to 7.9%. Finally, severe haemophiliacs at the beginning of the follow-up period amounted to 42%, with an observed decrease to 35% by the end of the follow-up period. The average age of adults (18 years and older) with haemophilia A was 42–45 years over the follow-up period. In 2013, 84% of patients (of any severity) received on-demand treatment, and 16% of patients received prophylaxis. In contrast, in 2021, 72% of patients received on-demand treatment, and 28% received prophylaxis. The high percentage of patients on on-demand treatment was influenced by the considerable proportion of patients with mild haemophilia A who did not have prophylaxis. The mean ABR was 5.3 bleeding episodes per year at the beginning and 0.8 bleeding episodes per year on average at the end, and the mean AJBR was 3.9 at the beginning and 0.5 at the end of the follow-up period. In contrast, the total consumption of coagulation factors increased by more than half over the follow-up period, which was also due to the increase in the number of patients on prophylaxis. At the start of the follow-up period, the average annual consumption of factor VIII concentrates was 1256 IU/kg/year, and at the end, it was 1928 IU/kg/year per treated patient during the year under review. Data associated with bleeding treatment and the necessity of hospitalisation have been available from the CNHP registry since 2018. In that year, there were 26 hospitalised cases due to bleeding (5.7%) amongst patients with haemophilia A; in 2021, there were 14 patients (3.0%) with haemophilia A. Compared to 2018, the average length of hospitalisation due to bleeding for all adult patients with haemophilia A decreased from an average of 15 hospitalisation days to 13. Data associated with the number of surgical procedures (all types) have been available in the registry since 2016. In that year, 36 (8.3%) adult patients with haemophilia A underwent surgical procedures. At the end of the observed period in 2021, there were 42 (8.9%) adult patients with haemophilia A.

**Table 1:** Patients’ characteristics and treatment outcomes: adult patients with haemophilia A

	2012, *N* = 358	2013, *N* = 377	2014, *N* = 402	2015, *N* = 431	2016, *N* = 436	2017, *N* = 441	2018, *N* = 453	2019, *N* = 469	2020, *N* = 471	2021, *N* = 470
Age										
Mean (SD)	42 (17)	42 (17)	42 (17)	43 (17)	44 (17)	44 (17)	44 (17)	44 (17)	45 (17)	45 (17)
Median (range)	40 (19–90)	39 (19–91)	39 (19–92)	40 (19–93)	41 (19–94)	41 (19–95)	41 (19–96)	41 (19–89)	42 (19–90)	43 (19–91)
Severity, *n* (%)										
Mild	159 (45)	178 (48)	196 (49)	221 (51)	230 (53)	234 (53)	245 (54)	251 (54)	258 (55)	261 (56)
Moderate	39 (11)	41 (11)	42 (10)	42 (9.7)	36 (8.3)	36 (8.2)	39 (8.6)	39 (8.4)	41 (8.8)	37 (7.9)
Severe	149 (42)	148 (40)	158 (39)	160 (37)	161 (37)	163 (37)	161 (36)	165 (36)	160 (34)	163 (35)
Inhibitor	4 (1.1)	5 (1.3)	6 (1.5)	8 (1.9)	9 (2.1)	8 (1.8)	8 (1.8)	8 (1.7)	8 (1.7)	7 (1.5)
Treatment regimen, *n* (%)										
OD	314 (88)	315 (84)	324 (81)	342 (79)	344 (79)	338 (77)	348 (77)	349 (74)	348 (74)	340 (72)
PX	44 (12)	62 (16)	78 (19)	89 (21)	92 (21)	103 (23)	105 (23)	120 (26)	123 (26)	130 (28)
ABR										
Mean (SD)	*NA*	5.3 (12.2)	4.7 (9.7)	3.3 (7.7)	3.0 (8.3)	2.5 (6.6)	1.8 (4.6)	1.6 (5.0)	1.2 (3.7)	0.8 (2.7)
Median (range)		0 (0–124)	0 (0–55)	0 (0–59)	0 (0–60)	0 (0–54)	0 (0–39)	0 (0–51)	0 (0–44)	0 (0–24)
JABR										
Mean (SD)	*NA*	3.9 (11.6)	3.6 (8.5)	2.3 (6.4)	2.2 (7.1)	1.8 (5.4)	1.1 (3.3)	1.0 (3.5)	0.8 (3.1)	0.5 (2.1)
Median (range)		0 (0–124)	0 (0–55)	0 (0–59)	0 (0–60)	0 (0–49)	0 (0–39)	0 (0–50)	0 (0–44)	0 (0–22)
Total of FVIII consumption (IU/kg per year)										
Mean (SD)	1264 (1300)	1256 (1880)	1486 (1958)	1273 (1396)	1579 (2136)	1475 (1746)	1781 (2509)	1845 (1669)	1875 (1772)	1928 (1911)
Median (IQR)	857 (274–1907)	731 (223–1844)	990 (318–2048)	802 (190–1961)	859 (226–2308)	780 (175–2293)	1163 (161–2917)	1555 (345–2952)	1707 (179–3168)	1533 (183–3152)
Surgery, *n* (%)	*NA*	*NA*	*NA*	*NA*	36 (8.3)	38 (8.6)	39 (8.6)	65 (13.9)	42 (8.9)	42 (8.9)
Hospitalisation due to bleeding, *n* (%)	*NA*	*NA*	*NA*	*NA*	*NA*	*NA*	26 (5.7)	22 (4.7)	24 (5.1)	14 (3.0)
Length of hospit. due to bleeding (days)										
Mean (SD)	*NA*	*NA*	*NA*	*NA*	*NA*	*NA*	15 (28)	12 (15)	18 (50)	13 (11)
Median (IQR)							7 (4–12)	5 (2–17)	7 (6–11)	10 (3–22)

*NA* not available, *ABR* annual bleeding rate, *JABR* joint annual bleeding rate, *OD* on demand, *PX* prophylaxis, *SD* standard deviation, *IQR* interquartile range

### Group of adult patients with severe haemophilia A in the CNHP registry

At the beginning of the follow-up period (2013) (Table 2), there were 148 adult patients with severe haemophilia A, and at the end of this period (2021), there were 163, with an average age of 41–44 years. Sixty-three percent of patients were on on-demand treatment at the beginning of the follow-up period, and 37% were on prophylaxis. Over time, this ratio gradually changed so that at the end of the follow-up period, only 26% of patients were receiving treatment on-demand, and 74% of patients with severe haemophilia A were on prophylaxis. The mean ABR fell from the original 12.4 to 1.8 at the end of the follow-up period. The average frequency of joint bleeding decreased from 11.3 to 1.3 at the end of the follow-up period. Of the 123 patients treated at the beginning, with a mean consumption of 1665 IU/kg/year, 97 (84%) were treated with plasma-derived factor VIII and 26 (22%) with recombinant factor VIII. At the end of the follow-up period, 163 patients (mean consumption of 2805 IU kg/year) were followed-up, of whom only 23 (16%) patients were treated with plasma-derived factor VIII, 60 (42%) were treated with recombinant factor VIII and 93 (65%) were treated with recombinant factor VIII with extended half-life (EHL) products (from only treated patients—143). Some patients were treated with multiple types of factor VIII concentrates within one year (this was always the year of switching from plasma products to recombinant/recombinant EHL). Since 2018, there were 15 (9.3%) hospitalised cases due to bleeding amongst patients with severe haemophilia A; in 2021, there were 4 patients (2.5%) with severe haemophilia A. Compared to 2018, the average length of hospitalisation due to bleeding for all adult patients with severe haemophilia A decreased from an average of 19 hospitalisation days to 2, which is up to 9.5 times less. This led to a substantial reduction in indirect costs associated with the implementation of prophylaxis. Since 2016, there were 16 (9.9%) adult patients, and at the end of the observed period in 2021, there were 11 (6.7%) adult patients with severe haemophilia A who underwent surgical procedures. The number of patients initially increased, as expected, due to planned surgeries being conducted after transitioning to prophylaxis, but in recent years, the numbers of surgical procedures have decreased again (Table 2). The most substantial decrease in the number of ABR and JABR events, as well as the number of hospitalisations due to bleeding and their duration, occurred at the end of the observed period when the number of patients with severe haemophilia A treated with recombinant FVIII-EHL increased by 65%.

**Table 2:** Patients’ characteristics and treatment outcomes: adult patients with severe haemophilia A

	2012, *N* = 149	2013, *N* = 148	2014, *N* = 158	2015, *N* = 160	2016, *N* = 161	2017, *N* = 163	2018, *N* = 161	2019, *N* = 165	2020, *N* = 160	2021, *N* = 163
Age										
Mean (SD)	40 (16)	41 (16)	42 (16)	42 (16)	43 (16)	43 (16)	43 (16)	43 (16)	44 (16)	44 (17)
Median (range)	38 (19–73)	39 (19–74)	40 (19–75)	40 (19–76)	41 (19–77)	40 (19–78)	40 (19–79)	40 (19–80)	42 (19–81)	41 (19–82)
Treatment regimen, *n* (%)										
OD	116 (78)	93 (63)	88 (56)	79 (49)	76 (47)	66 (40)	65 (40)	52 (32)	46 (29)	42 (26)
PX	33 (22)	55 (37)	70 (44)	81 (51)	85 (53)	97 (60)	96 (60)	113 (68)	114 (71)	121 (74)
ABR										
Mean (SD)	*NA*	12.4 (16.9)	10.9 (13.1)	7.6 (10.9)	7.3 (12.2)	6.1 (9.8)	4.5 (6.9)	4.2 (7.8)	2.7 (5.6)	1.8 (4.2)
Median (range)		6 (0–124)	5 (0–55)	3 (0–59)	2 (0–60)	2 (0–54)	2 (0–39)	1 (0–51)	1 (0–44)	0 (0–24)
JABR										
Mean (SD)	*NA*	11.3 (18.0)	8.8 (12.2)	5.7 (9.7)	5.8 (10.8)	4.5 (8.2)	2.9 (5.2)	2.7 (5.5)	1.9 (4.9)	1.3 (3.3)
Median (range)		4 (0–124)	4 (0–55)	2 (0–59)	1 (0–60)	1 (0–49)	1 (0–39)	1 (0–50)	0 (0–44)	0 (0–22)
Total of FVIII consumption (IU/kg per year)										
Mean (SD)	1572 (1378)	1665 (2154)	1865 (2186)	1742 (1432)	2050 (1909)	1935 (1613)	2237 (1612)	2509 (1535)	2665 (1597)	2805 (1588)
Median (IQR)	1219 (531–2143)	1330 (480–2195)	1402 (651–2305)	1467 (674–2453)	1749 (699–2904)	1690 (567–2972)	2095 (582–3317)	2472 (1512–3405)	2601 (1704–3733)	2836 (1747–4105)
pdFVIII therapy, *n* (%)	62 (82)	97 (84)	105 (79)	93 (73)	94 (67)	94 (64)	74 (51)	62 (42)	32 (23)	23 (16)
rFVIII therapy, *n* (%)	15 (20)	26 (22)	28 (21)	33 (26)	51 (36)	65 (44)	81 (56)	96 (65)	68 (49)	60 (42)
rFVIII EHL therapy, *n* (%)	0 (0)	0 (0)	0 (0)	0 (0)	0 (0)	0 (0)	0 (0)	37 (25)	62 (45)	93 (65)
Surgery, *n* (%)	*NA*	*NA*	*NA*	*NA*	16 (9.9)	18 (11)	17 (10.6)	20 (12.1)	15 (9.4)	11 (6.7)
Hospitalisation due to bleeding, *n* (%)	*NA*	*NA*	*NA*	*NA*	*NA*	*NA*	15 (9.3)	10 (6.1)	13 (8.1)	4 (2.5)
Length of hospit. due to bleeding (days)										
Mean (SD)	*NA*	*NA*	*NA*	*NA*	*NA*	*NA*	19 (37)	4 (4)	7 (5)	2 (1)
Median (IQR)							7 (4–11)	2 (1–6)	7 (2–10)	2 (1–3)

*NA* not available, *ABR* annual bleeding rate, *JABR* joint annual bleeding rate, *OD* on demand, *PX* prophylaxis, *pdFVIII* plasma-derived factor VIII, *rFVIII* recombinant factor VIII, *EHL* extended half-life, *SD* standard deviation, *IQR* interquartile range

### Group of adult patients with severe haemophilia A according to therapeutic regimen

Patients were categorised into two groups based on the therapeutic regimen in the follow-up years: the prophylaxis group and the on-demand group throughout the follow-up period. Transitional prophylaxis, which corresponded to years in which there was a transition from OD to PX or vice versa, was excluded from the analysis. Patients on prophylaxis were followed for an average of 5.7 years, whilst patients on the on-demand regimen were followed for 5 years (Table 3). Patients on prophylaxis had fewer bleeding episodes than on-demand patients (ABR 2.1 vs. 7.4, *p* = 0.003; AJBR 1.3 vs. 6.3, *p* = 0.005). A coefficient adjusted from GLMM or the age of patients showed a 66% decrease in all bleeding episodes (0.34 (95% Cl 0.29–0.4), *p* < 0.001), including joint bleeding episodes (0.34 (0.28–0.41), *p* < 0.001), in prophylaxis patients. The total mean consumption of factor VIII concentrates in prophylaxis patients was 3195 IU/kg/year (of which the mean consumption of plasma factor VIII was 2149 IU/kg/year, that of recombinant factor VIII was 2913 IU/kg/year and that of recombinant EHL factor VIII was 3125 IU/kg/year); in on-demand treatment, the total mean consumption of FVIII concentrate was 732 IU/kg/year (of which the mean consumption of plasma factor VIII was 622 IU/kg/year, that of recombinant factor VIII was 808 IU/kg/year and that of recombinant EHL factor VIII was 514 IU/kg/year).

**Table 3:** Patients’ characteristics and treatment outcomes according to the therapeutic regimen (adult patients with severe haemophilia A)

	Prophylaxis, *N* = 63	On demand, i = 74	OD + PX, *N* = 106	*p* value^1^
Age at start of follow-up (years)				< 0.001
Mean (SD)	28 (13)	46 (16)	37 (14)	
Median (range)	22 (19–69)	48 (19–73)	34 (19–71)	
Follow-up duration (years)				0.23
Mean (SD)	5.7 (3.5)	5.0 (3.5)	7.9 (2.5)	
Median (range)	6 (1–10)	4 (1–10)	9 (2–11)	
Sum	359	373	835	
PX dose (IU/kg per week)				–
Mean (SD)	62 (21)	*NA*	50 (19)	
Median (IQR)	61 (45–77)		48 (37–58)	
ABR				0.003
Mean (SD)	2.1 (2.1)	7.4 (9.5)	6.9 (7.5)	
Median (range)	1.5 (0.0–8.0)	2.6 (0.0–44.0)	4.2 (0.0–36.5)	
JABR				0.005
Mean (SD)	1.3 (1.6)	6.3 (9.2)	4.6 (6.4)	
Median (range)	0.7 (0.0–5.5)	2.0 (0.0–44.0)	2.5 (0.0–32.1)	
Total of FVIII consumption (IU/kg per year)				< 0.001
Mean (SD)	3195 (1118)	732 (903)	2205 (1320)	
Median (IQR)	3150 (2414–4064)	465 (225–754)	1909 (1377–2816)	
Total of pdFVIII consumption (IU/kg per year)				< 0.001
Mean (SD)	2149 (966)	622 (698)	1817 (2417)	
Median (IQR)	2129 (1569–2523)	455 (225–709)	1287 (887–2072)	
Total of rFVIII consumption (IU/kg per year)				< 0.001
Mean (SD)	2913 (1105)	808 (1022)	2338 (1359)	
Median (IQR)	2827 (2346–3579)	424 (157–914)	2068 (1500–2717)	
Total of rFVIII EHL consumption (IU/kg per year)				< 0.001
Mean (SD)	3125 (1100)	514 (401)	2057 (1001)	
Median (IQR)	3196 (2349–3932)	562 (327–726)	2092 (1371–2616)	

*NA* not available, *ABR* annual bleeding rate, *JABR* joint annual bleeding rate, *OD* on demand, *PX* prophylaxis, *pdFVIII* plasma-derived factor VIII, *rFVIII* recombinant factor VIII, *EHL* extended half-life, *SD* standard deviation, *IQR* interquartile range

^1^Wilcoxon rank sum test; comparison of prophylaxis vs. on-demand groups

### Prophylaxis effect in patients with severe haemophilia A with a change in therapeutic regimen during follow-up

This part of the analysis focused on the comparison of the prophylaxis effect in a group of 106 patients with severe haemophilia A who had both on-demand and prophylactic regimens during follow-up (Table 4). During the on-demand regimen, the mean total ABR in these patients was 11.8, and the mean AJBR was 9. For prophylaxis, the average total ABR in the same patients was 3.4, and the AJBR was 2.3 (both *p* < 0.001). The average on-demand consumption of factor VIII concentrates was 1500 IU/kg/year, whereas with a prophylactic regimen, it was 2676 IU/kg/year (*p* < 0.001).

**Table 4:** Comparison of treatment results on PX and OD regimens in patients with both regimens during the observed period (adult patients with severe haemophilia A)

	OD, *N* = 106	PX, *N* = 106	*p* value^1^
Age at start of treatment regimen (years)			
Mean (SD)	37.6 (13.9)	39.6 (14.9)	
Median (range)	34.5 (26.2–47.8)	37.0 (28.0–49.0)	
Follow-up duration (years)			0.003
Mean (SD)	3.3 (2.2)	4.6 (2.7)	
Median (range)	3 (1–9)	4 (1–9)	
Sum	350	485	
PX dose (IU/kg per week)			
Mean (SD)	*NA*	50.2 (21.2)	
Median (IQR)		47.4 (37.3–59.2)	
ABR			< 0.001
Mean (SD)	11.8 (12.7)	3.4 (5.8)	
Median (range)	8.2 (0.0–57.0)	1.9 (0.0–36.5)	
JABR			< 0.001
Mean (SD)	9.0 (12.4)	2.3 (4.2)	
Median (range)	5.0 (0.0–54.5)	1.0 (0.0–28.3)	
ABR, *n* (%)			< 0.001
0–1	22 (26)	41 (40)	
2–5	12 (14)	41 (40)	
> 5	52 (60)	20 (20)	
JABR, *n* (%)			< 0.001
0–1	29 (37)	50 (51)	
2–5	12 (15)	40 (40)	
> 5	38 (48)	9 (9.1)	
Total of FVIII consumption (IU/kg per year)			< 0.001
Mean (SD)	1500 (1591)	2676 (1291)	
Median (IQR)	1165 (646–1692)	2489 (1891–3091)	
Total of pdFVIII consumption (IU/kg per year)			0.002
Mean (SD)	1595 (2619)	2027 (1370)	
Median (IQR)	1050 (620–1599)	1837 (1273–2518)	
Total of rFVIII consumption (IU/kg per year)			< 0.001
Mean (SD)	1184 (871)	2529 (1387)	
Median (IQR)	1286 (346–1706)	2312 (1538–3059)	
Total of rFVIII EHL consumption (IU/kg per year)			0.95
Mean (SD)	1555 (751)	2054 (1057)	
Median (IQR)	1404 (1062–2120)	2159 (1348–2740)	

*NA* not available, *ABR* annual bleeding rate, *JABR* joint annual bleeding rate, *OD* on demand, *PX* prophylaxis, *pdFVIII* plasma-derived factor VIII, *rFVIII* recombinant factor VIII, *EHL* extended half-life, *SD* standard deviation, *IQR* interquartile range

^1^Wilcoxon signed rank test with continuity correction

## Discussion

**Fig. 1:**
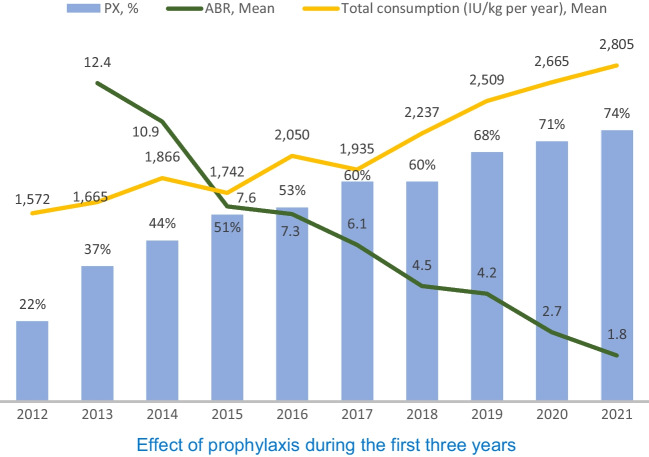
Effect of prophylaxis during the first three years. Legend: PX = prophylaxis, ABR = annual bleeding rate

Over the reported period, there was a considerable decrease in the total number of joint bleeding episodes in prophylaxis patients compared to those receiving on-demand treatment. Total ABR in patients with severe haemophilia A decreased 6.9-fold during the follow-up period, and AJBR decreased up to 8.7-fold. Our results correlate with the results of the POTTER study [4], in which ABR and AJBR in the prophylactic regimen were reduced 7- to eightfold compared to on-demand treatment. Patients in our registry who were on prophylaxis or on-demand regimens (Table 3) showed a decrease in ABR of 3.5 and AJBR of 4.85-fold, but it needs to be highlighted treated on-demand was less than half of that in the POTTER study. In the publication by Collins et al. [5], the number of joint bleeding episodes on prophylaxis even decreased from a median of 15 in on-demand regimen to a median of 0 on prophylaxis, and the number of total bleeding episodes decreased from a median of 20.5 to a median of 0 on prophylaxis (20–40 IU/kg 3 times a week) at the documented trough level FVIII of 4–6%, which cannot be compared with our patients who did not have a trough level monitored, and prophylaxis treatment was targeted to achieve a trough level above 1–2% according to the CNHP recommendation from 2017 [6]. Miesbach et al. [7] stated that prophylaxis had the greatest effect on the reduction of bleedings in patients of all ages, both in patients with severe haemophilia A but also in those with moderate and mild forms of the disease (median ABR 22.1 in patients with severe haemophilia treated only on-demand compared to patients treated only prophylactically—median 1.5). In patients with severe haemophilia A who switched from on-demand to prophylaxis, the total ABR decreased from 38 to 1.1. In our follow-up (Table 3), the observed decrease was less pronounced, but our patients treated on-demand had documented bleeding that was much less (approximately 1/3 of the published ABR). The greatest effect of prophylaxis was observed during the first three years (Fig. 1), when prophylaxis was available only to patients with a very high number of bleeding episodes. The proportion of patients on prophylaxis increased by 29% (from 22% in 2012 to 51% in 2015, equal to 2.32 times), and consumption increased only slightly (by 11%). However, overall consumption has been increasing since the introduction of prophylaxis for younger patients whose previous bleeding rate was not as high. In contrast, the total consumption of coagulation factors increased by more than 2/3 over the follow-up period in the CNHP registry, which was also caused by the increase in the number of patients treated for prophylaxis. Our evaluation is also consistent with a recent short-term prospective study confirming the efficacy of secondary prophylaxis in reducing bleeding rates, including the data from the first year of the randomised three-year SPINART study [8]. It can also be compared with the study by Valentino et al. [9], which, in addition to the comparison of the two prophylactic regimens, also deals with the comparison of the effect of the number of joint bleeding episodes in patients initially treated on-demand for 6 months (ABR = 43.9) and then on prophylaxis for 12 months (ABR = 1.1); however, our patients had 2.5- to 3.5-fold less bleeding with the on-demand regimen compared to the SPINART and Valentino studies, and the median ABR in our patients was 1.5 and 1.9, respectively (Tables 3 and 4). The Austria Haemophilia Registry [10] records show that from 2012 to 2017, the ABR for spontaneous bleeding was 22.0 with on-demand treatment vs. an ABR of 4.1 with prophylaxis in patients with severe haemophilia A. Similar results were also published in a European retrospective study [11] that included European centres/registries. The median ABR for patients on prophylaxis ranged from 1.0 to 4.0 for severe haemophilia A, and the median ABRs for on-demand-treated severe haemophilia A ranged from 4.5 to 18.0. Our nine-year assessment of the effect of prophylaxis shows the effect of minimal joint bleeding, especially in patients without previous more severe joint impairment, but the question of whether there will be long-term beneficial effects on joint outcomes in patients with proven joint damage remains unanswered. The positive impact of prophylaxis on the reduction of symptoms of haemophilic arthropathy should be considered as an additional benefit of tertiary prophylaxis in patients with evidence of joint damage. These clinical benefits of tertiary prophylaxis may be an incentive to adhere to this treatment regimen, although they are inevitably associated with a significantly higher consumption of FVIII concentrate against treatment on-demand. Long-term implementation of tertiary prophylaxis entails higher costs related to currently expected longer life expectancy in patients with haemophilia as well as the increased risk of bleeding caused by ageing and comorbidities [12]. On the other hand, as the data from the registry showed, there was a significant decrease in hospitalisations due to bleeding and their duration. This significantly reduced the indirect expenditures associated with the introduction of prophylaxis. The additional parameters related to indirect treatment expenditures (i.e. days lost from work or school) were not monitored in the CNHP registry. We have been tracking the joint scores of patients in recent years when the majority of severe haemophilia A patients were on prophylaxis, so our results cannot be compared with those derived from data at the beginning of the registry when most patients were treated on-demand. Prophylaxis can be assumed to decrease and reduce other costs, especially for demanding orthopaedic surgery, whilst increasing the quality of life of patients with haemophilia [13]. It needs to be mentioned that there will be other factors contributing to an increase in the consumption of FVIII concentrates. Historically, a target trough level above 1% seems insufficient for the minimum ABR level, and now the recommended factor VIII level above 3% is based on the recommendation of the EDAM (European Directorate for the Quality of Medicines & Health Care 5/2020).

## Conclusion

In the Czech Republic registry, during the gradual transition of patients with severe haemophilia A from on-demand treatment to prophylaxis, a clear reduction in the number of bleeding episodes over the follow-up period was observed. Total ABR in all patients with severe haemophilia A decreased 6.9-fold, AJBR decreased 8.7-fold, and factor VIII consumption increased by 68.5%. In a subgroup of patients with severe haemophilia A who had switched from an on-demand to a prophylactic regimen, total ABR decreased 3.5-fold, and AJBR decreased 3.9-fold. The total consumption of FVIII concentrates increased by 78.4%. We can assume that the continuation of prophylaxis, especially in severe haemophiliacs, may prevent further damage to joints, and in some patients, it could delay demanding orthopaedic surgery, as well as other bleeding episodes, especially those that may be fatal for patients without prophylaxis.

## Data Availability

https://www.cnhp.cz/index.php?pg=registr--vysledky.
